# Cost of Severe Chronic Obstructive Pulmonary Disease Exacerbations in a High Burden Region in North India

**DOI:** 10.5334/aogh.2423

**Published:** 2019-01-22

**Authors:** Parvaiz A. Koul, Aqsa Amin Nowshehri, Umar H. Khan, Rafi A. Jan, S. U. Shah

**Affiliations:** 1 Departments of Internal and Pulmonary Medicine and Geriatric Medicine, Sheri Kashmir Institute of Medical Sciences, Srinagar, IN; 2 Sheri Kashmir Institute of Medical Sciences, Srinagar, IN

## Abstract

**Background:**

Data on costs of acute exacerbations of COPD (AECOPD) in low-income countries are sparse. We conducted a prospective survey to assess direct and indirect costs of severe AECOPD in a tertiary care setting in a high prevalence area of North India.

**Methods:**

We conducted face-to-face surveys using a semi-structured questionnaire among a convenience sample of 129 consenting patients admitted with AECOPD. Data were collected on out-of-pocket costs of hospitalization, consultation, medications, diagnostics, transportation, lodging, and missed work days for self and their attendants. Out-of-pocket costs were supplemented with World Health Organization-CHOICE estimates. Missed work-days were valued on per capita national income (Indian Rupees [INR] 68,748, US$1,145.8). Median total cost per exacerbation episode was INR 44,390 (Inter-quartile range [IQR]: INR 33,354–63,642; US$739.8, IQR: 555.9–1060.7). Hospital costs constituted the largest component of the costs (71%) followed by other costs directly borne by the patient himself (29%), medicine costs (14%), transportation charges (2%) and diagnostic tests (3%). Indirect costs to caregivers (median INR 1,544, IQR: INR 0–17,370 INR; US$25.7, IQR: US$0–289.5), calculated as financial loss due to missed work days, accounted for 4% of the total cost. Expenses were covered by family members in all but 11 patients.

**Conclusions:**

AECOPD in India are associated with substantial costs and strategies to reduce the burden of disease such as smoking cessation, influenza and pneumococcal vaccination, etc should be aggressively pursued.

## Introduction

COPD is a disease characterized by non-fully reversible airflow limitation and a progressive decline in lung function. According to the World Health Organization (WHO), 274 million people died from COPD in 2000, making it the third major cause of morbidity and mortality globally [1,2,3]. Presentations of COPD include an exacerbation of respiratory symptoms (AECOPD), ranging from mild to severe and often requiring hospital admission [4,5,6]. Exacerbations can lead to increased morbidity and mortality, thus placing an individual patient at higher risk of future exacerbations [7]. AECOPD are more common with increasing severity of disease: 50% of the patients with severe disease experienced at least two exacerbations per year [8,9]. Prior studies have also shown that COPD envisages tremendous costs to patients and the health care system; and exacerbations augment this cost substantially [10].

With population of 1.2 billion, India is second only to China in population and is expected to surpass China by 2025. India has a high prevalence of COPD (4% compared to 0.9% in males and 0.7% in women worldwide) and contributes to more than 20% of all COPD deaths globally [2,11,12]. The prevalence of COPD in Kashmir, located in northern India, is even higher amounting to about 19% [Koul et al, unpublished data].

While many studies have estimated the burden of COPD, there is limited research about the costs of AECOPD. A Canadian study that enrolled 790 COPD patients with an exacerbation (151 requiring hospitalization) estimated an average cost of US$9,557 [13]. Similarly, a Swedish study concluded that the cost of moderate and severe COPD exacerbations were SEK 2,111 and SEK 21,852, respectively [14]. Another Swedish cohort of 2,414 patients found that the mean cost of an AECOPD was US$159, though the costs were determined based on severity [15]. However, these studies were conducted in developed countries. The only study from India was limited to direct costs, and the study estimated that the cost per exacerbation was US$89.75 [6]. The goal of the present study is to calculate both the direct and indirect cost incurred as a result of missed work by the patient himself or his/her caregivers as a result of a COPD exacerbation.

## Methods

### Setting

This observational study was conducted between September 2012 to April 2013 in the Department of Internal and Pulmonary Medicine of SKIMS, a 650-bed tertiary care hospital located in Srinagar, India. All the patients who were admitted to the hospital with a diagnosis of severe AECOPD were enrolled in the study.

### Definition of Severe COPD

Severe AECOPD was defined as worsening of respiratory symptoms beyond expected daily variability, leading to respiratory failure requiring hospital admission. All enrolled individuals provided informed consent.

### Data Collection

Semi-structured questionnaires were administered at admission, discharge, and two weeks after discharge. During the interviews, we collected data on out-of-pocket cost of admission, consultation, medications, diagnostic investigations, transportation, lodging fees, and number of missed work days of both patients and caregivers. A structured questionnaire was also administered by a medical social worker at admission and discharge to the patient’s themselves or their health care providers. Additional information about changes in the treatment plan during hospitalization was collected from the treating physician and nurse. The questionnaire also collected information about patients’ sociodemographic characteristics.

### Valuation of Costs

Direct costs were defined as the cost of admission, consultation, medications, diagnostics (e.g. radiologic and laboratory studies), transportation, and lodging fees. Direct cost components were self-reported during in-person or telephone follow-up interviews. WHO-CHOICE estimates for outpatient and hospital-bed day cost were used to supplement data on self-reported direct medical costs [17]. The 2008 estimates were adjusted to 2012 using the consumer price index for India [18].

Indirect cost was defined as the monetary value of lost wages of adult patients and caregivers of all age groups due to inability to perform regular employment duties. Estimation of indirect cost assumed that labor was replaced at a cost to maintain societal productivity [20].

Direct out-of-pocket patient costs were calculated as the sum of the direct medical costs and direct non-medical costs (transportation during the current hospitalization and follow-up visits to health facilities during the same episode). Total direct out-of-pocket cost of hospitalization at public facilities was supplemented with WHO-CHOICE estimates of hospital-bed day cost and the median length of hospital stay [17]. The total direct cost of hospitalization or outpatient visit at public facilities was then calculated as the sum of total patients’ out-of-pocket direct cost and the estimated cost to the government based on WHO-CHOICE data. Due to inconsistencies in self-reported data, the number of patient missed work days were estimated by adding two days to the reported length of hospital stay [19]. Indirect costs were estimated as the product of missed work days and the 2012 per capita income per day [20].

Medians and interquartile ranges are reported because data were not normally distributed. The exchange rate used for conversion of INR to US$ was 1 INR = US$0.016 [21]. Median costs were compared to monthly or annual per capita income (INR 5,790 per month or INR 68,408 per year) [22].

## Results

### Patient Characteristics

A total of 129 patients and their caregivers were enrolled in the study. The mean age of the patients was 68 ± 10 years and 54% were male. The median duration of hospital stay was 8 days with a range of 1–24 days; inpatient mortality was 12%. Since the study was conducted in a public hospital, 21% of patients did not have to pay for tests carried during the hospitalization. Almost half (49%) of study subjects were transferred from other hospitals. Additionally, 110 (85%) caregivers lost days of work taking care of their family member (median 9.5 days, range 0–90 days). The 12 (9%) patients who took sick leave had to remain off their duties for a median of 20 days (range 5–30 days).

The median cost of hospitalization was INR 44,390 per admission with an interquartile range (IQR): INR 33,354–63,642. The percentage attributed to each cost component are shown in Table 1. Cost incurred to the hospital was highest (71%) followed by the cost directly borne by patients (29%), costs of medicines (14%), transportation charges (2%), and diagnostic charges including blood chemistries and imaging (3%). Indirect costs calculated as financial loss due to missed work days to caregivers accounted for 4% of the total cost. The median indirect cost was INR 1,544 (IQR: 0–17,370). The median cost of discharge medications was INR 700 (IQR: INR 500–900). The expenses incurred due to AECOPD were covered by family members in all but 11 patients.

**Table 1:** Costs Related to Chronic Obstructive Pulmonary Disease Exacerbations.

Cost Category	Median INR	IQR

**Pre-hospitalization**	1500	0–1500
**Medications**	5800	3600–9000
**Diagnostic-related**	550	40–1500
**Direct loss to patients as a result of missed work**	2123	0–0
**Hospital charges**	330	250–480
**Transportation**	900	450–1720
**Financial loss to relatives**	1544	965–2316
**Discharge medications**	700	500–900
**Hospital costs per patient**	31500	24500–45000
**Patient total** **(direct and indirect)**	13195	8985–19360
**Total**	44390	33354–63642

IQR: Interquartile Range.

## Discussion

While considerable data is available about risk factors and burden of AECOPD, there is limited information about costs of AECOPD, particularly from developing countries. Our study estimated the costs of AECOPD in India to be INR 44,390 per admission and mostly (71%) related to direct hospital costs. Importantly, about 30% of the total costs were related to transportation, medications and diagnostic tests and out of pocket expenses. These results suggest that AECOPD generates substantial costs that could be a major economic burden to patients and their families.

According to the NCMH, the estimated economic burden of COPD in India in 2005 was 35,000 crore rupees (Rs. 350 billion or US$5.8 billion) and is estimated to further increase in the future [23]. Multiple studies have consistently shown that acute exacerbations are major contributors to these costs, ranging from 45–70% of costs [24,25,26]. NCMH also showed that the major component of expenses was incurred on managing exacerbations (average cost of exacerbation US$174). The only other study conducted in India estimated the total cost of a hospitalized exacerbation to be US$90 [16].

One of the largest surveys assessing the economic impact of COPD was conducted in Europe and North America. That study found that nearly 40–57% of direct medical COPD costs are a result of AECOPD-related hospitalizations. These costs are substantially higher in patients with severe disease [27]. Montegi also found high expenses related to inpatient care for AECOPD in Japan with a mean cost of US$8,214 [28]. A meta-analysis of eleven studies conducted between 1998–2008 estimated costs of exacerbations to vary widely across different countries from US$88 to US$7,757 per exacerbation; the largest component was related to hospitalization and strongly correlated with the severity of exacerbation [29].

The population of the state of Jammu & Kashmir, located in northern India is close to 12 million. (Census 2011, http://www.census2011.co.in/census/state/jammu+and+kashmir.html) Given an expected prevalence of COPD of 19% in individuals over 40 years of age, we can estimate that there are approximately 470 thousand COPD patients in these states. Patients with severe (GOLD stage III or IV) COPD have a rate of exacerbations of 3.43 events per year and those with moderate (GOLD II) COPD have an average of 2.68 events per year [30]. Assuming that at least half of COPD patients will have two exacerbations per year, we can estimate that approximately 2.1 billion Indian rupees are spent on AECOPD in Jammu and Kashmir.

In the present study, the inpatient mortality due to AECOPD was 12%. Prior studies have reported mortality rates from 11 to 24% [31,32,33]. Not only is AECOPD associated with high mortality, but exacerbations have a significant negative impact on health-related quality of life. Connors showed that at 6months post AECOPD, 54% of patients required assistance with at least one activity of daily living and 49% considered their health status to be fair or poor [33]. Additionally, it has been estimated that COPD contributes to 28,000 disability adjusted life years (DALYs) worldwide with a sizeable proportion being from patients in the South East Asian region, particularly India [34]. However, under-reporting of AECOPD is common leading to underestimation of the true burden of COPD-related costs [35].

India bears a disproportionate burden of COPD morbidity and mortality not only because of the size of the population, but also because of under-treatment. In addition, given limitations in insurance coverage, most COPD care cost is borne by the patients or their caregivers. Thus, appropriate management of COPD may require national programs to provide quality care to those patients that could not otherwise afford healthcare. While similar programs are currently available for malaria and tuberculosis, COPD is rapidly growing as a major source of morbidity and mortality in India [36].

In summary, our study estimated the cost of AECOPD in northern India and found that a considerable proportion of costs are covered by patients and/or their family members. These findings could help hospital administrators and policymakers plan for the care of patients with this growing chronic disease.

## References

[1] Global Initiative for Chronic Obstructive Lung Disease (GOLD). Global Strategy for the Diagnosis, Management and prevention of Chronic Obstructive Pulmonary Disease; 2013 http://www.goldcopd.org/uploads/users/files/GOLD_Report_2013_Feb20.pdf. Accessed April 27, 2014.

[2] Lopez AD, Shibuya K, Rao C, Mathers CD, Hansell AL, Held LS, et al. Chronic obstructive pulmonary disease: current burden and future projections. Eur Respir J. 2006; 27(2): 397–412. DOI: 10.1183/09031936.06.00025805 16452599

[3] Mathers CD and Loncar D. Projections of global mortality and burden of disease from 2002 to 2030. PLoS Med. 2006; 3(11): e442 DOI: 10.1371/journal.pmed.0030442 17132052 PMC1664601

[4] Wedzicha JA and Seemungal TA. COPD exacerbations: Defining their cause and prevention. Lancet. 2007 Sep. 1; 370(9589): 786–96. 17765528 10.1016/S0140-6736(07)61382-8 PMC7134993

[5] Soler-Cataluña JJ, Martínez-García MA, Román Sánchez P, Salcedo E, Navarro M and Ochando R. Severe acute exacerbations and mortality in patients with chronic obstructive pulmonary disease. Thorax. 2005; 60(11): 925–31. DOI: 10.1136/thx.2005.040527 16055622 PMC1747235

[6] Bakerly ND, Davies C, Dyer M and Dhillon P. Cost analysis of an integrated care model in the management of acute exacerbations of chronic obstructive pulmonary disease. Chron Respir Dis. 2009; 6(4): 201–8. DOI: 10.1177/1479972309104279 19729444

[7] Garcia-Aymerich J, Pons IS, Mannino DM, Maas AK, Miller DP and Davis KJ. Lung function impairment COPD hospitalisations and subsequent mortality. Thorax. 2011; 66(7): 585–90. DOI: 10.1136/thx.2010.152876 21515553

[8] Hurst JR, Vestbo J, Anzueto A, Locantore N, Müllerova H, Tal-Singer R, et al. Susceptibility to exacerbation in chronic obstructive pulmonary disease. N Engl J Med. 2010; 363(12): 1128–38. DOI: 10.1056/NEJMoa0909883 20843247

[9] Garcia-Aymerich J, Farrero E, Félez MA, Izquierdo J, Marrades RM and Antó JM. Risk factors of readmission to hospital for a COPD exacerbation: a prospective study. Thorax. 2003; 58(2): 100–5. DOI: 10.1136/thorax.58.2.100 12554887 PMC1746561

[10] Pasquale MK, Sun SX, Song F, Hartnett HJ and Stemkowski SA. Impact of exacerbations on health care costs and resource utilization in chronic obstructive pulmonary disease patients with chronic bronchitis from a predominantly medicare population. Int J Chron Obstruct Pulmon Dis. 2012; 7: 757–764. DOI: 10.2147/COPD.S36997 23152680 PMC3496536

[11] WHO. Global Infobase. https://apps.who.int/infobase/Index.aspx. Accessed March 10, 2012.

[12] Jindal SK, Aggarwal AN, Gupta D, Agarwal R, Kumar R, Kaur T, et al. Indian study on epidemiology of asthma, respiratory symptoms and chronic bronchitis in adults. Int J Tuberc Lung Dis. 2012; 16(9): 1270–7. DOI: 10.5588/ijtld.12.0005 22871327

[13] Mittmann N, Kuramoto L, Seung SJ, Haddon JM, Bradley-Kennedy C, Fitzgerald JM, et al. The cost of moderate and severe COPD exacerbations to the Canadian healthcare system. Res Med. 2008; 102(3): 413–21. DOI: 10.1016/j.rmed.2007.10.010 18086519

[14] Andersson F, Borg S, Jansson SA, Jonsson AC, Ericsson A, Prütz C, et al. The cost of exacerbations in chronic obstructive pulmonary disease (COPD). Respir Med. 2002; 96(9): 700–8. DOI: 10.1053/rmed.2002.1334 12243316

[15] Miravitlies M, Murio C, Guerrero T, Gisbert R and DAFNE Study Group. Pharmacoeconomic evaluation of acute exacerbation of chronic bronchitis COPD. Chest. 2002; 121(5): 1449–55. DOI: 10.1378/chest.121.5.1449 12006427

[16] Veettil SK, Ma S, Rajiah K and Kumar S. Cost of acute exacerbation of COPD in patients attending government hospital in Kerala, India. Int J of Pharmacy and Pharmaceutical Sciences. 2012; 4(3): 659–61.

[17] WHO. WHO-CHOICE Unit Cost Estimates 2007–2008. http://www.who.int/choice/country/country_specific/en/index.html. Accessed April 27, 2014.

[18] Government of India Labour Bureau. http://labourbureau.nic.in/indnum.html.

[19] Molinari NM, Ortega-Sanchez IR, Messonnier ML, Thompson WW, Wortley PM, Weintraub E, et al. The annual impact of seasonal influenza in the US: Measuring disease burden and costs. Vaccine. 2007 Jun. 28; 25(27): 5086–96. 17544181 10.1016/j.vaccine.2007.03.046

[20] Press Trust of India. India’s per capita income rises to Rs 5,729 per month Business Standard 2 7, 2013 http://www.business-standard.com/article/economy-policy/india-s-per-capita-income-rises-to-rs-5-729-per-month-113020700995_1.html.

[21] US Dollar (USD) to Indian Rupee (INR) exchange rates. http://www.exchangerates.org.uk/USD-INR-exchange-rate-history.html.

[22] Press Trust of India. India’s per capita income rises to Rs 5,729 per month Business Standard 2 7, 2013 http://www.business-standard.com/article/economy-policy/india-s-per-capita-income-rises-to-rs-5-729-per-month-113020700995_1.html.

[23] Murthy KJR and Sastry JG. Economic burden of chronic obstructive lung disease, National commission on macroeconomics and health burden of disease in India. 2005; 264–271.

[24] Hilleman DE, Dewan N, Malesker M and Friedman M. Pharmacoeconomic evaluation of COPD. Chest. 2000; 118(5): 1278–85. DOI: 10.1378/chest.118.5.1278 11083675

[25] Sullivan SD, Ramsey SD and Lee TA. The economic burden of COPD. Chest. 2000; 117(2 Suppl.): 5S–9S. DOI: 10.1378/chest.117.2_suppl.5S 10673466

[26] Dalal AA, Liu F and Riedel AA. Cost trends among commercially insured and Medicare Advantage-insured patients with chronic obstructive pulmonary disease: 2006 through 2009. Int J Chron Obstruct Pulmon Dis. 2011; 6: 533–42. DOI: 10.2147/COPD.S24591 22069365 PMC3206770

[27] Dahl R and Lofdahl CG. The economic impact of COPD in North America and Europe. Analysis of the Confronting COPD survey. Introduction. Respir Med. 2003; 97(Suppl C): S1–2. 10.1016/s0954-6111(03)80019-7 12647937

[28] Motegi T, Yamada K and Kida K. Cost analysis for inpatient therapy for patients with acute exacerbations of chronic obstructive pulmonary disease. Nihon Kokyuki Gakkai Zasshi. 2006; 44(11): 787–94. 17144574

[29] Toy EL, Gallagher KF, Stanley EL, Swensen AR and Duh MS. The economic impact of exacerbations of chronic obstructive pulmonary disease and exacerbation definition: A review. COPD. 2010; 7(3): 214–28. DOI: 10.3109/15412555.2010.481697 20486821

[30] Donaldson GC, Seemungal TAR, Bhowmik A and Wedzicha JA. Relationship between exacerbation frequency and lung function decline in chronic obstructive pulmonary disease. Thorax. 2002; 57(10): 847–852. DOI: 10.1136/thorax.57.10.847 12324669 PMC1746193

[31] Paggiaro PL, Dahle R, Bakran I, et al. Multicentre randomized placebo-controlled trial of inhaled fluticasone propionate in patients with chronic obstructive pulmonary disease. Lancet. 1998; 351: 773–780. DOI: 10.1016/S0140-6736(97)03471-5 9519948

[32] Seneff MG, Wagner DP, Wagner RP, et al. Hospital and 1-year survival of patients admitted to intensive care units with acute exacerbation of chronic obstructive pulmonary disease. JAMA. 1995; 274: 1852–7. DOI: 10.1001/jama.1995.03530230038027 7500534

[33] Connors AF, Jr., Dawson NV, Thomas C, et al. Outcomes following acute exacerbation of severe chronic obstructive lung disease. The SUPPORT investigators (Study to Understand Prognoses and Preferences for Outcomes and Risks of Treatments). Am J Respir Crit Care Med. 1996; 154: 959–967. DOI: 10.1164/ajrccm.154.4.8887592 8887592

[34] Shibuya K, Mathers CD and Lopez AD. Chronic Obstructive Pulmonary Disease (COPD): Consistent estimates of incidence, prevalence and mortality by WHO region. Global Programme on Evidence for Health Policy, World Health Organization; 2001.

[35] Langsetmo L, Platt RW, Ernst P and Bourbeau J. Underreporting exacerbation of chronic obstructive pulmonary disease in a longitudinal cohort. Am J Respir Crit Care Med. 2008; 177(4): 396–401. DOI: 10.1164/rccm.200708-1290OC 18048806

[36] Bhome AB. COPD in India: Iceberg or volcano. J Thorac Dis. 2012; 4(3): 298–309. 22754670 10.3978/j.issn.2072-1439.2012.03.15 PMC3378191

